# Polysulfobetaines in Aqueous Solution and in Thin Film Geometry

**DOI:** 10.3390/ma11050850

**Published:** 2018-05-21

**Authors:** Bart-Jan Niebuur, Jonas Puchmayr, Christian Herold, Lucas P. Kreuzer, Viet Hildebrand, Peter Müller-Buschbaum, André Laschewsky, Christine M. Papadakis

**Affiliations:** 1 Physik-Department, Fachgebiet Physik weicher Materie/Lehrstuhl für Funktionelle Materialien, Technische Universität München, 85748 Garching, Germany; bart.niebuur@ph.tum.de (B.-J.N.); jonas.puchmayr@tum.de (J.P.); christian.herold@ph.tum.de (C.H.); lucas.kreuzer@ph.tum.de (L.P.K.); muellerb@ph.tum.de (P.M.-B.); 2 Institut für Chemie, Universität Potsdam, 14476 Potsdam-Golm, Germany; viethildebrand@gmx.de (V.H.); Laschews@uni-potsdam.de (A.L.); 3 Fraunhofer Institut für Angewandte Polymerforschung, Geiselbergstr. 69, 14476 Potsdam-Golm, Germany

**Keywords:** polyzwitterions, polysulfobetaines, dynamic light scattering, phase behavior

## Abstract

Polysulfobetaines in aqueous solution show upper critical solution temperature (UCST) behavior. We investigate here the representative of this class of materials, poly (*N*,*N*-dimethyl-*N*-(3-methacrylamidopropyl) ammonio propane sulfonate) (PSPP), with respect to: (i) the dynamics in aqueous solution above the cloud point as function of NaBr concentration; and (ii) the swelling behavior of thin films in water vapor as function of the initial film thickness. For PSPP solutions with a concentration of 5 wt.%, the temperature dependence of the intensity autocorrelation functions is measured with dynamic light scattering as function of molar mass and NaBr concentration (0–8 mM). We found a scaling of behavior for the scattered intensity and dynamic correlation length. The resulting spinodal temperatures showed a maximum at a certain (small) NaBr concentration, which is similar to the behavior of the cloud points measured previously by turbidimetry. The critical exponent of susceptibility depends on NaBr concentration, with a minimum value where the spinodal temperature is maximum and a trend towards the mean-field value of unity with increasing NaBr concentration. In contrast, the critical exponent of the correlation length does not depend on NaBr concentration and is lower than the value of 0.5 predicted by mean-field theory. For PSPP thin films, the swelling behavior was found to depend on film thickness. A film thickness of about 100 nm turns out to be the optimum thickness needed to obtain fast hydration with H_2_O.

## 1. Introduction

Water-soluble thermo-responsive polymers belong to the class of stimuli-sensitive polymeric systems (often referred to as “smart materials”), which can undergo dramatic changes of their properties in response to small changes of temperature [1,2,3]. In most aqueous thermo-responsive polymer solutions, a reversible coil-globule collapse transition of the macromolecules takes place at the phase separation temperature [4]. A lower critical solution temperature (LCST) or upper critical solution temperature (UCST) behavior may be encountered, depending on whether the miscibility gap occurs at high or low temperatures.

A number of studies have addressed the solution behavior of thermo-responsive polymers having lower critical solution temperature (LCST) behavior [3,5,6]. At low temperatures, these are well-hydrated by water molecules and water-soluble, whereas they collapse at higher temperatures [4], release water and form new H-bonds, which may be intra- or interchain type [5,7]. The collapsed chains form compact globules which aggregate, causing turbidity in the solution [8] or a shrinkage in thickness of thin films [9].

**Figure 1:**
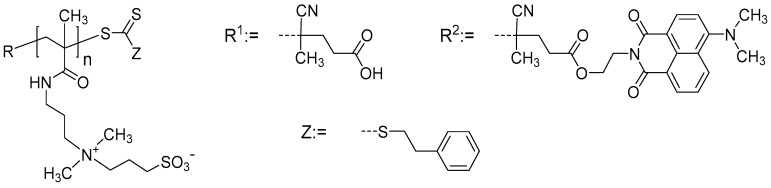
Chemical structure of the polyzwitterions under investigation, PSPP_n_ [18]. R^2^ serves as label for future UV-Vis spectroscopy. For R^1^, the degree of polymerization is n = 80, while for R^2^, n = 85 and 280.

Vice versa, in the case of polymers exhibiting UCST behavior, the polymer chains undergo a phase transition from an expanded well-hydrated state to a collapsed and mostly dehydrated state upon cooling [10,11,12]. Such UCST behavior has been found rarely in aqueous polymer solutions, but it is often encountered for polyzwitterions bearing sulfobetaine moieties [13,14]. This polymer class is particularly attractive because it is well known for a high biocompatibility [15,16]. One representative of this class of polymers, namely poly (*N*,*N*-dimethyl-*N*-(3-methacrylamidopropyl) ammonio propane sulfonate) (PSPP, Figure 1), was studied in detail with somewhat peculiar findings [17,18,19]. While being rather insensitive to the nature of the end groups R and Z, the cloud point (CP) of PSPP depends not only markedly on its molar mass and on the use of either H_2_O or D_2_O (strong H–D isotope effect), but is also very sensitive to the type and concentration of added low molar mass salts, in a non-linear and complex fashion [18,19]. The strongest effects on its CP were typically found for chaotropic anions [18,19] in agreement with the Hofmeister series [20,21], increasing, e.g., in the order SO_4_^2−^ < Cl^−^ < Br^−^. Remarkably, the effect of salts may be non-monotonous; at low salt concentrations, the CP of PSPP increases with the amount of added salt and passes through a maximum, beyond which it continuously decreases. For NaBr, the maximum was found at about 3 mM for 5 wt.% PSPP_500_ solutions in H_2_O [18]. In these studies, the CP was determined as function of, among others, molar mass and NaBr content using turbidimetry; however, no further characterization was attempted. 

Besides polymers in solution, especially thermo-responsive polymer films receive very high attention as an advanced device in numerous application fields because of their characteristic phase transition behavior [22,23,24,25]. Analogous to aqueous PSPP solutions, PSPP thin films can undergo a phase transition upon a temperature increase above their UCST, which is manifest through an absorption of water molecules that is followed by a swelling of the film. This process is completely reversible, as the film re-collapses to its initial film thickness upon a temperature decrease below the UCST. For implementing such thin films in application fields such as nano-sensors/switches, soft-robotic and artificial pumps and muscles, a controlled sorption and diffusion of gaseous and liquid penetrants into the polymeric network is of essential importance. The analysis of the underlying mechanism of the sorption and release of low-molecular penetrants and the polymer-water interaction in general, but also the equilibrium structure close to the interfaces (substrate and air), is crucial for the devices that are based on a fast and reliable penetration of small molecules [22,23,24]. 

In the present study, we investigate the behavior of aqueous solutions and thin films of three PSPP_n_ samples, differing in molar mass (number average degrees of polymerization n = 80–280). For the solutions, we chose a polymer concentration of 5 wt.%, which is sufficiently high to give reasonable scattering signals and represents the semi-dilute regime. Moreover, we varied the NaBr concentration between 0 and 8 mM in H_2_O, i.e., around the salt concentration for which a maximum of CP was found previously [18]. Using dynamic light scattering (DLS), we determine the scattered intensity as well as the dynamic correlation length as function of molar mass, NaBr concentration and temperature. We then derived the spinodal temperatures from both quantities and determined the scaling exponents. 

Furthermore, we analyzed the swelling behavior of PSPP_80_ thin films of different thicknesses at a constant temperature. Thin films are prepared by spin-coating, and the film thickness is adjusted by the choice of the concentration of the polymer solution. By applying a theoretical swelling model [26,27,28], characteristic constants such as the effective Flory Huggins parameter χ_eff_ and the time constant τ_swell_ are obtained, which promote a better understanding of the mechanism behind the kinetic processes.

## 2. Results

### 2.1. Polymer Solutions

**Figure 2:**
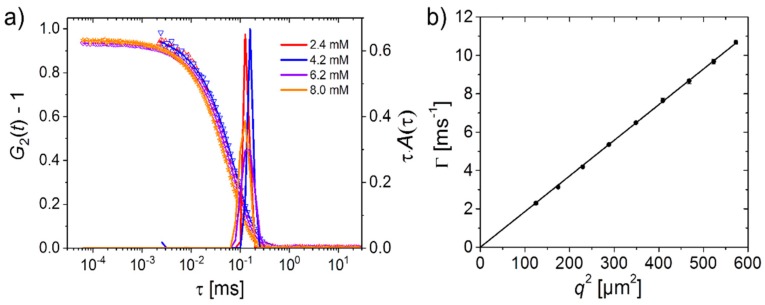
Representative results from DLS on a 5 wt.% solution of PSPP_280_ in H_2_O. (**a**) Left axis: Intensity autocorrelation functions at θ = 90° and 30 °C in relation to NaBr concentration, as indicated in the graph. The time ranges of the curves differ because they were measured on two different instruments, see the Materials and Methods Section. Right axis: Corresponding distribution functions of relaxation times in equal area representation, τA(τ) vs. log(τ); (**b**) Relaxation rate, Γ, of PSPP_280_ in 1.4 mM NaBr in H_2_O at 25.3 °C vs. *q*^2^. Symbols: measured data, line: linear fit through the origin.

DLS gives information about the polymer dynamics in the temperature range above the CP, where the PSPP_85_ and PSPP_280_ solutions investigated are optically clear [29]. Typical intensity autocorrelation functions G_2_(t) and the corresponding distribution functions of relaxation times τ are shown in Figure 2a, by the example of PSPP_280_. The correlation functions show a single decay which results in a single, relatively narrow peak in the distribution functions. Angle-dependent measurements reveal that the relaxation rate Γ = 1/τ is proportional to the square of the momentum transfer, *q*^2^ (Figure 2b), i.e., a diffusional process is observed. We attribute this process to concentration fluctuations in the polymer solution [30].

**Figure 3:**
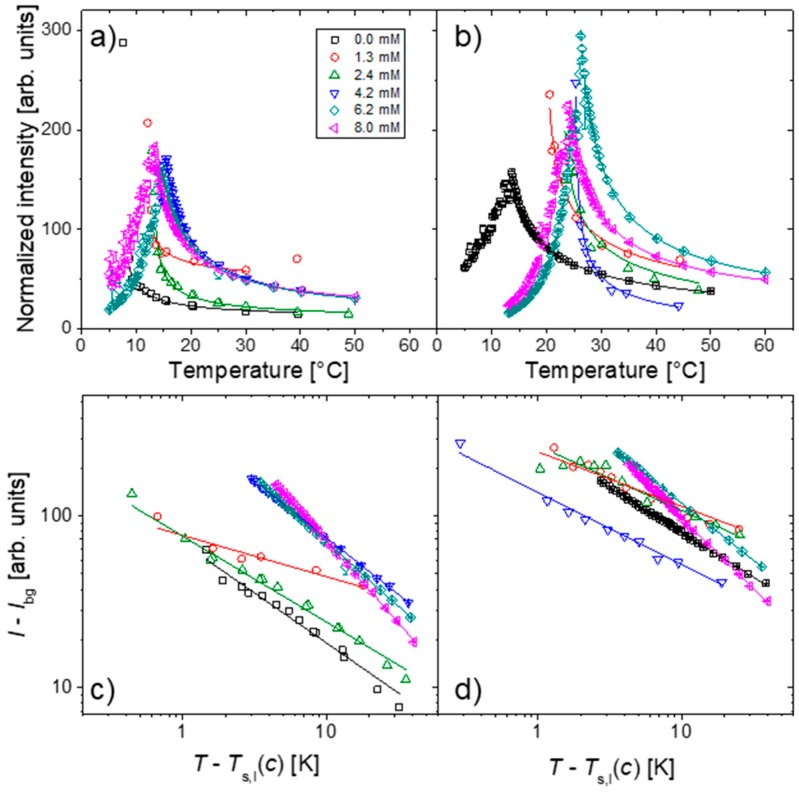
(**a**,**b**) Scattered intensities in relation to temperature; (**c**,**d**) Same intensity data in relation to reduced temperature, τ, in log-log representation. Symbols: experimental data as detailed in the legend of (**a**), lines: fits. (**a**,**c**) PSPP_85_, (**b**,**d**) PSPP_280_ at 5 wt.% in H_2_O and at the NaBr concentrations indicated. The intensities are normalized to the incoming flux. The temperature range of the lines corresponds to the data points used for the fits. In (**c**,**d**), only points from the one-phase state are plotted, i.e., at temperatures on the higher side of the intensity maximum.

The scattered intensity as function of temperature is shown in Figure 3. As the solutions are cooled towards their respective CP, the intensities increase significantly due to increasingly important concentration fluctuations, and reach a maximum. Below this temperature, the solutions become turbid, i.e., large aggregates form, and the scattered intensity decreases again due to multiple scattering. 

Fitting the intensities at high temperatures, i.e., in the one-phase state, with the expression [31].

(1) $$I=I_{0}{\left(\frac{T-T_{s,I}}{T_{s,I}}\right)}^{-\gamma }+I_{bg}$$

gives the spinodal temperature, *T*_s,I_, and the critical exponent of the susceptibility, γ. Within mean-field theory, the latter is expected at γ = 1.0 [31]. The fits are shown in Figure 3a,b. Plotting the data along with these fits in a log–log representation with respect to *T* − *T*_s,I_ (Figure 3c,d) shows that the fits are good down to *T* − *T*_s,I_ ≅ 1 K and that the intensities indeed display scaling behavior. 

**Figure 4:**
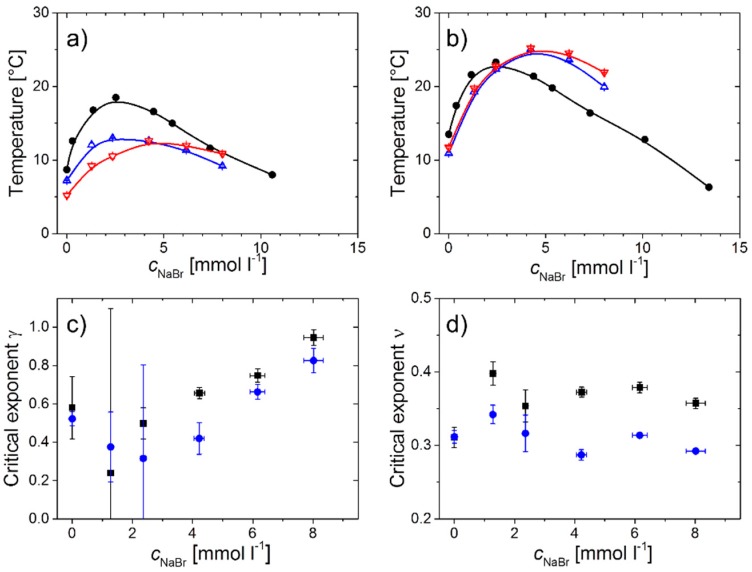
Cloud points measured by turbidimetry (black closed circles, [32]) and spinodal temperatures from fits of Equation (1) to the scattered intensity, *T*_s,I_ (blue open up triangles), and of Equation (2) to the dynamic correlation lengths, *T*_s,__ξ_ (red open down triangles). (**a**) PSPP_85_ and (**b**) PSPP_280_, both at 5 wt.% in H_2_O. The lines guide the eye. (**c**,**d**) Exponents γ from Equation (1) (**c**) and ν from Equation (2) (**d**). Black squares: PSPP_85_, blue circles: PSPP_280_.

The resulting *T*_s,I_ values are shown in Figure 4 along with the CPs from turbidimetry [32]. The latter were determined in cooling runs and were chosen as those temperatures where the light transmission was decreased by 5%. For both polymers, the CP values show non-monotonous behavior with NaBr concentration, *c*_NaBr_, featuring maxima at *c*_NaBr_ = 2.5 mM for PSPP_85_ and 2.4 mM for PSPP_280_. The *T*_s,I_ values show overall similar behavior, but deviate by a few kelvin from the CP values. From thermodynamics, it is expected for UCST-type polymer solutions, that the spinodal line (determined by extrapolation of the scattered intensity) is located at lower temperatures than the binodal line (measured by turbidimetry). For PSPP_85_, the *T*_s,I_ values are indeed a few kelvin lower than the CP values in the entire range of NaBr concentrations studies. Moreover, they feature a maximum at *c*_NaBr_ = 2.4 mM, i.e., at the same NaBr concentration as the CP values. In contrast, for PSPP_280_, the maximum of *T*_s,I_ is shifted to *c*_NaBr_ = 4.2 mM. Moreover, the *T*_s,I_ values are lower than the CP values only for values of c_NaBr_ up to 2.4 mM (which is actually the value where the CP is maximum), while they are larger at high values. The reason for the unexpected behavior at *c*_NaBr_ > 2.4 mM may be the difference in determination of the cloud points and the spinodal temperatures by turbidimetry and dynamic light scattering. Moreover, it may hint at a qualitatively different behavior of the PSPP_280_ solution beyond the maximum of the CP. Comparing the two polymers, it is seen that the spinodal temperature increases with the degree of polymerization of the polymers, as expected.

In salt-free H_2_O, the exponent γ is 0.58 and 0.52 for PSPP_85_ and PSPP_280_, respectively, i.e., below the value of one predicted by mean-field theory [31]. With increasing salt concentration, it decreases further and reaches minimum values at *c*_NaBr_ = 1.3 mM for PSPP_85_ (where γ = 0.24) and 2.4 mM for PSPP_280_ (where γ = 0.32). Above this value of *c*_NaBr_, it increases linearly and reaches the values of 0.97 and 0.83 for PSPP_85_ and PSPP_280_, respectively, at *c*_NaBr_ = 8.0 mM. Addition of sufficiently high amounts of NaBr thus renders the solutions more mean-field like. These findings may again hint at the origin of the maximum of the CP in dependence on *c*_NaBr_.

**Figure 5:**
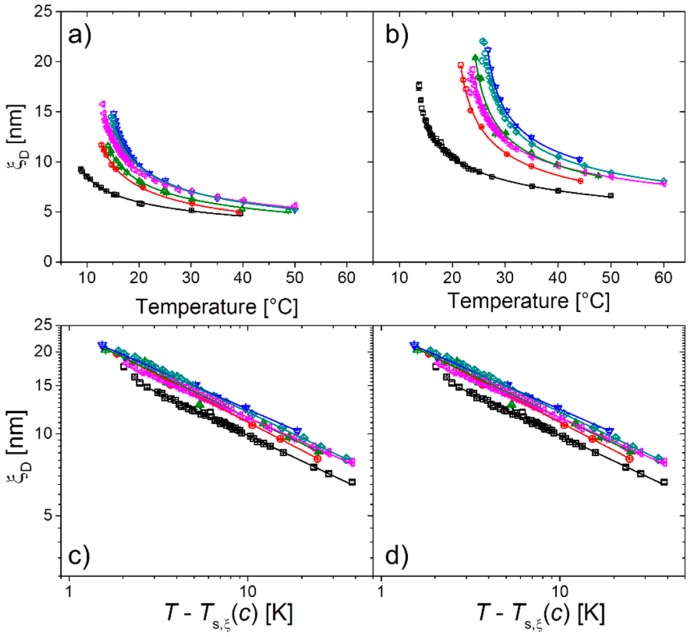
(**a**,**b**) Dynamic correlation lengths, ξ_D_, as function of temperature. The *T*-range of the lines corresponds to the data points used for the fits. (**c**,**d**) Same intensity data in dependence on reduced temperature, τ, in log-log representation. Symbols: experimental data as in Figure 3, lines: fits of Equation (2) (**a**,**c**) PSPP_85_, (**b**,**d**) PSPP_280_.

From the relaxation times measured at a scattering angle of 90°, the dynamic correlation length, ξ_D_, may be determined (Equation (3) and (4) below). We expect ξ*_D_* to follow scaling behavior as well:

(2) $$\xi_{D}=\xi_{0}{\left(\frac{T-T_{s,\xi }}{T_{s,\xi }}\right)}^{-\nu }$$

with a spinodal temperature, *T*_s,__ξ_, and the critical exponent of the correlation length, ν. The latter was predicted by mean-field theory to be 0.5 [31]. The values of ξ*_D_* decrease from ~16 nm to 4.5 nm and from ~26 to ~6.6 nm for PSPP_85_ and PSPP_280_, respectively; thus, they are higher for the higher molar mass polymer. The fits of Equation (2) are good, indicating that scaling behavior is indeed observed (Figure 5a,b). The ξ*_D_* values along with the fits are plotted in Figure 5c,d in a log–log representation as function of reduced temperature. The data lie on straight lines, proving that Equation (2) is appropriate, except, in some cases, very close to *T*_s,__ξ_. The resulting critical temperatures, *T*_s,__ξ_, differ slightly from *T*_s,I_ for PSPP_85_, but are very similar for PSPP_280_ (Figure 4a,b). The scaling exponents ν are independent of *c*_NaBr_ (Figure 4d) and take values of ν = 0.36 ± 0.03 for PSPP_85_ and 0.31 ± 0.02 for PSPP_280_. Both values are lower than the one predicted by mean-field theory. 

### 2.2. Thin Films

**Figure 6:**
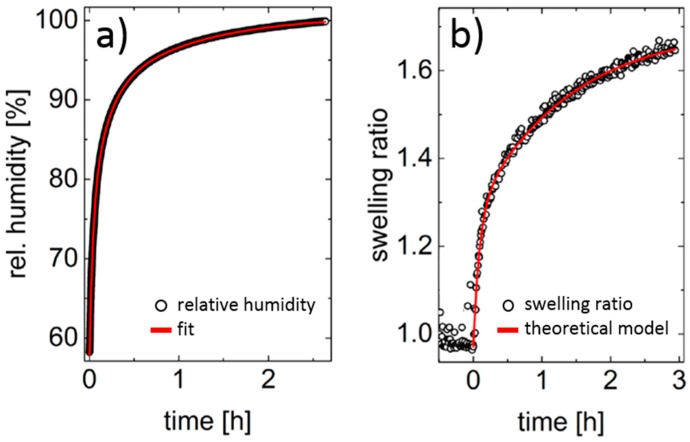
(**a**) Evolution of the relative humidity inside the vapor chamber. The curve is fitted with an exponential function; (**b**) Swelling ratio of the PSPP_80_ film upon increasing relative humidity at 12 °C measured in-situ with SR. The data are fitted by a theoretical model, see text.

Due to the absence of crosslinks, thin films are not directly exposed to water, but to water vapor. The swelling behavior of PSPP_80_ thin films during exposure to an atmosphere of high humidity is analyzed in-situ using spectroscopic reflectometry (SR). A custom-made vapor chamber allows for control of temperature and relative humidity. Figure 6a shows the ambient relative humidity inside the hydration chamber at 12 °C. Since the CP of a 5 wt.% solution is at 12 °C, and the concentration dependence is rather flat in this concentration range, we expect that at the measuring temperature of 12 °C, the film is in the one-phase state, i.e., above the UCST expected at high polymer concentration. Swelling of the film is induced by injecting H_2_O inside the chamber, which corresponds to *t* = 0. The film thickness starts to increase strongly as a function of time directly after water injection, as evident in the swelling ratio (Figure 6b). A quantitative description of the swelling process of the PSPP_80_ thin film is achieved by applying a theoretical model that respects both the intrinsic swelling kinetics of the films and also the varying relative humidity [26,27,28]. In this way, characteristic parameters such as the time constant τ_swell_ of the swelling kinetics and the effective Flory Huggins parameter χ_eff_ can be determined. A more detailed explanation of the swelling model is given below. 

The experiment was repeated for four PSPP_80_ films with different film thicknesses in the range 30–117 nm. The observed swelling ratios as a function of time are fitted with the swelling model (Equation (6) below), and the values obtained for χ_eff_ and τ_swell_ are compiled in Table 1.

## 3. Discussion

Solutions of PSPP_85_ and PSPP_280_ (5 wt.% in H_2_O) were investigated using DLS near the clearing point as function of NaBr concentration in a range of 0–8 mM. A single, diffusive process is observed, which is attributed to concentration fluctuations in the polymer solution. At high temperatures, the intensities and the dynamic correlation lengths feature scaling behavior. The critical exponent of the susceptibility is 0.52–0.58 in salt-free solution, i.e., lower than the mean-field value of 1.0. It increases with *c*_NaBr_ and reaches 0.8–1.0 at c_NaBr_ = 8.0 mM. The critical exponent of the correlation length takes values of 0.31–0.36, independent of *c*_NaBr_, which is lower than the value predicted by mean-field theory (0.5). The spinodal temperatures obtained from fits of the two scaling laws nearly coincide with each other. They are higher for the longer polymer, as expected. For PSPP_85_, the spinodal temperatures are lower than the cloud points measured by turbidimetry [32], as expected from the usual phase behavior of polymer solutions. The same holds for PSPP_280_, but only for c_NaBr_ up to 2.4 mM, while the spinodal temperatures are unexpectedly larger than the CPs at higher *c*_NaBr_ values. This is possibly related to the non-linear behavior of the CP with *c*_NaBr_. 

The evolution of film thickness during increasing relative humidity was measured with SR and was evaluated with a theoretical model that gives information about the hydrophilicity of the polymer through the effective interaction parameter χ_eff_. Furthermore, the time constant τ_swell_ is obtained that gives details about the time scales of the swelling process. Overall, four PSPP_80_ thin films, varying in initial film thickness (30–117 nm) were analyzed. The highest interaction parameter χ_eff_ was found for the thinnest polymer film, indicating a rather hydrophobic behavior. This is corroborated by the highest time constant τ_swell_ (242 s) for this film. This demonstrates that the diffusion of H_2_O molecules inside the polymer film—and thus its saturation during the swelling process—are slow. With increasing film thickness, the effective Flory Huggins parameter first decreases (χ_eff_ = 0.74 for a film thickness of 99 nm), indicating a more hydrophilic behavior of the polymer film, before it increases again (χ_eff_ = 0.78 and 0.82 for film thicknesses of 104 nm and 117 nm, respectively). Accordingly, the time constant first decreases strongly (τ_swell_ = 37 s for a film thickness of 99 nm) and increases again with increasing film thickness (τ_swell_ = 76 s and 81 s for film thicknesses of 104 nm and 117 nm, respectively). Thus, it seems that, for a fast hydration, an intermediate film thickness is optimum. While thinner films are rather dominated by interface effects, in thicker films, bulk behavior has a stronger impact on the swelling behavior. In addition, residual solvent might also have a contribution. Penetration of water into films containing trifluoroethanol may have been easier due to H-bonding with trapped solvent and greater chain mobility in the thicker samples. The findings mean that both interface- and bulk-dominated thin films are not hydrated as rapidly as thin films with a well-balanced ratio between interfaces and bulk. In turn, the film thickness can be used as control parameter for tailoring the speed of the film swelling. It should be noted that a significantly faster film swelling is expected for thin films exposed to water instead of water vapor [33].

## 4. Materials and Methods 

The sample characteristics are given in Table 2. Details of the polymer synthesis by the RAFT (radical addition-fragmentation chain transfer) technique [34] using both non-labeled and dye-labeled trithiocarbonate chain transfer agents and their molecular characterization were described in Ref. [18]. The RAFT process enables not only the preparation of polymers with predefined molar masses and low polymer dispersity, but also the incorporation of well-defined end groups [35]. While the sample for the thin film studies was made using a standard RAFT agent (R^1^, see Figure 1 [36]), the samples for solution studies were synthesized using a functionalized RAFT agent that bears a naphthalimide–dye (R^2^, see Figure 1 [18,19]), which enables complementary molecular characterization of the polyzwitterions by UV/vis and fluorescence spectroscopy [18,19,37]. Figure 1 displays the chemical structure of the resulting polymers PSPP_n_ along with the Z and the respective R end groups.

Samples for DLS were prepared by dissolving the appropriate amounts of PSPP_85_ or PSPP_280_ in NaBr solutions in demineralized water (0 to 8.0 mM). These solutions were heated to 20–40 °C and were shaken to dissolve the polymer. They were filtered using filters with pore sizes of 5 µm and 0.45 µm. 

Two setups were used for dynamic light scattering (DLS). The first setup consisted of an ALV-5000/E correlator (ALV-Laser Vertriebsgesellschaft mbH, Langen, Germany) together with a goniometer with an index-matching vat filled with toluene and kept at 24 °C by a JULABO F32 thermostat (JULABO Labortechnik GmbH, Seelbach, Germany). The light source was a HeNe laser operated at 35 mW and λ = 632.8 nm. The scattered light was measured using an ALV/SO-SIPD photomultiplier to which the signal was fed by an optical fiber. The second setup was a LS Spectrometer (LS Instruments, Fribourg, Switzerland) featured a HeNe laser with a maximum output power of 21 mW. The laser intensity could be adjusted. The sample was immersed in a decalin bath. The sample temperature was controlled by a JULABO CF31 thermostat and was kept at 24 °C. In both instruments, the sample was mounted in a cylindrical cuvette.

With both instruments, a scattering angle θ of 90° was chosen for the cooling runs. Ten to twenty measurements with a duration of 60 s each were carried out at each temperature. In single cases, angle-dependent measurements at fixed temperatures were carried out as well.

The measured intensity autocorrelation functions, *G*_2_(τ), were analyzed by inverse Laplace transformation (ILT) using the routine REPES [38], which calculated the distribution function of relaxation times, τA(τ) vs. log(τ). The mean relaxation times of each mode were extracted as the centers of mass of the peaks. The diffusion coefficients were calculated from the dependence of the decay rates

(3) $$\Gamma =\frac{1}{\tau }=Dq^{2}$$

on the momentum transfer, *q* = 4π*n* sin(θ/2)/λ where *n* is the temperature-dependent refractive index of water. The dynamic correlation lengths, ξ*_D_*, were calculated using the Stokes–Einstein relation,

(4) $$\xi_{D}=\frac{k_{B}T}{6\pi \eta D}$$

where *k_B_* is Boltzmann’s constant, *T* the absolute temperature and η the temperature-dependent viscosity of water.

For measurements by SR, silicon substrates were cleaned in an acid bath consisting of 54 mL H_2_O, 84 mL H_2_O_2_ and 198 mL H_2_SO_4_ for 15 min at 80°C in order to remove any organic substances from the surface. Possible traces of the acid bath were removed by rinsing the substrate for at least 10 min. Subsequently, the Si substrates were exposed to an oxygen plasma for 10 min in order to install a hydrophilic surface. PSPP_80_ films were prepared by spin-coating (2500 rpm, 15 min) the polymer out of a trifluoroethanol solution at room temperature. By applying solvent vapor annealing treatment (15 min) in saturated trifluoroethanol directly after spin-coating, a polymer surface of high homogeneity was generated. For SR measurements, a Filmetrics F20 thin-film analyzer (Filmetrics Inc., San Diego, CA, USA) together with a custom-made temperature-controlled humidity chamber was used. The bottom part of the chamber comprised an elevated platform where the sample was mounted. The sample stage was surrounded by a channel that was filled with water by injecting it through a permeable membrane on the top part of the chamber. The temperature of the whole setup was controlled by a JULABO F12 MC thermal bath, pumping water with the desired temperature through tubes in the walls of the chamber. The sample was illuminated with white light (λ = 400–1100 nm) through a glass window on top. In addition, a sensor was installed near the sample to track relative humidity and temperature during the hydration experiments.

The resulting swelling ratios in dependence on time were evaluated using to a humidity-sensitive model [26,27,28]. This model considers the intrinsic swelling kinetics, which is in principle driven by the diffusion of H_2_O molecules into the polymer film. This effect can be expressed by:

(5) $$\frac{d\left(t\right)}{d_{0}}=\frac{d_{\max}}{d_{0}}-\left(\frac{d_{\max}}{d_{0}}-1\right)Be^{-\frac{t}{\tau_{\mathrm{swell}}}}$$

where *d*_0_ is the initial film thickness of the dry polymer film and *d*(*t*) is the measured film thickness at time *t*. *d*_max_/*d*_0_ represents the maximum swelling ratio which the PSPP_80_ film is theoretically able to reach for a given ambient relative humidity. τ_swell_ is the time constant of the diffusion-driven swelling process, and *B* is a fitting parameter. The diffusion-driven, intrinsic swelling process usually limits the swelling in the early stages of the swelling process. However, the fact that the relative humidity is not constant (Figure 6a) has to be taken into account. The equilibrium sorption of the PSPP_80_ film at a given relative humidity *p*/*p*_sat_ of H_2_O atmosphere is described by the regular solution theory according to:

(6) $$ln\left(\frac{p}{p_{sat}}\right)=\ln\left(1-\frac{d_{0}}{d_{max}}\right)+\left(1-\frac{V_{H_{2}O}}{V_{\mathrm{PSPP}}}\right)\frac{d_{0}}{d_{max}}+\chi_{\mathrm{eff}}{\left(\frac{d_{0}}{d_{max}}\right)}^{2}$$

*V*_H2O_ and *V*_PSPP_ stand for the molar volumes of water and the polymer, respectively. χ_eff_ is the effective Flory Huggins parameter between the polymer and the water molecules, which can also be seen as a measure for the intermixing of both components or the hydrophilicity of the PSPP_80_ film.

## Figures and Tables

**Table 1:** Values of the fitting parameters χ_eff_ and τ_swell_ for the thin films from PSPP_80_ with H_2_O obtained by SR.

*d*_0_ [nm]	χ_eff_	τ_swell_ [s]
30 nm	0.89 ± 0.01	242 ± 11
99 nm	0.74 ± 0.01	37 ± 5
104 nm	0.78 ± 0.01	76 ± 2
117 nm	0.82 ± 0.01	81 ± 5

**Table 2:** Sample characteristics of polyzwitterions PSPP_n_ studied: number-average molar mass *M*_n_, degree of polymerization *DP*_n_ and cloud point CP.

Sample	*M*_n_ (kg mol^−1^) ^a^	*DP*_n_^a^	CP (°C) ^b^
PSPP_80_^c^	24	80	12
PSPP_85_^d^	26	85	8.7
PSPP_280_^d^	82	280	13.0

**^a^** Calculated from the molar ratio of monomer/chain transfer agent and the conversion determined by ^1^H NMR spectroscopy. **^b^** Determined by turbidimetry for 5 wt.% solutions in Millipore water (H_2_O) during cooling runs at a cooling rate of 0.2 K min^-1^. **^c^** End groups R^1^ and Z. **^d^** End groups R^2^ and Z (Figure 1), values for *M*_n_ and CP taken from Ref. [18].
